# *In vivo* gene transfer targeting in pancreatic adenocarcinoma with cell surface antigens

**DOI:** 10.1186/1476-4598-11-81

**Published:** 2012-10-22

**Authors:** Marie Lafitte, Benoit Rousseau, Isabelle Moranvillier, Miguel Taillepierre, Evelyne Peuchant, Véronique Guyonnet-Dupérat, Aurélie Bedel, Pierre Dubus, Hubert de Verneuil, François Moreau-Gaudry, Sandrine Dabernat

**Affiliations:** 1 INSERM U1035, Bordeaux, France; 2 Université Bordeaux Segalen, Bordeaux, France; 3 Animalerie A2, Université Victor Segalen, Bordeaux, France; 4 Vectorology platform, Université Bordeaux Segalen, Bordeaux, France; 5 Université Bordeaux Segalen INSERM U1035, 146, rue Léo Saignat, Bordeaux Cedex, 33076, France

**Keywords:** Pancreatic adenocarcinoma, Targeted therapy, Surface marker

## Abstract

**Background:**

Pancreatic ductal adenocarcinoma is a deadly malignancy resistant to current therapies. It is critical to test new strategies, including tumor-targeted delivery of therapeutic agents. This study tested the possibility to target the transfer of a suicide gene in tumor cells using an oncotropic lentiviral vector.

**Results:**

Three cell surface markers were evaluated to target the transduction of cells by lentiviruses pseudotyped with a modified glycoprotein from Sindbis virus. Only Mucin-4 and the Claudin-18 proteins were found efficient for targeted lentivirus transductions *in vitro*. In subcutaneous xenografts of human pancreatic cancer cells models, Claudin-18 failed to achieve efficient gene transfer but Mucin-4 was found very potent. Human pancreatic tumor cells were modified to express a fluorescent protein detectable in live animals by bioimaging, to perform a direct non invasive and costless follow up of the tumor growth. Targeted gene transfer of a bicistronic transgene bearing a luciferase gene and the herpes simplex virus thymidine kinase gene into orthotopic grafts was carried out with Mucin-4 oncotropic lentiviruses. By contrast to the broad tropism VSV-G carrying lentivirus, this oncotropic lentivirus was found to transduce specifically tumor cells, sparing normal pancreatic cells *in vivo*. Transduced cells disappeared after ganciclovir treatment while the orthotopic tumor growth was slowed down.

**Conclusion:**

This work considered for the first time three aspect of pancreatic adenocarcinoma targeted therapy. First, lentiviral transduction of human pancreatic tumor cells was possible when cells were grafted orthotopically. Second, we used a system targeting the tumor cells with cell surface antigens and sparing the normal cells. Finally, the TK/GCV anticancer system showed promising results *in vivo*. Importantly, the approach presented here appeared to be a safer, much more specific and an as efficient way to perform gene delivery in pancreatic tumors, in comparison with a broad tropism lentivirus. This study will be useful in future designing of targeted therapies for pancreatic cancer.

## Background

Pancreatic ductal adenocarcinoma (PDAC) is a highly malignant disease and is the fourth cause of death from cancer in the western world. Due to the lack of specific symptoms, the diagnosis is delayed and PDAC are commonly detected at advanced stages of the disease. Regardless of the treatment, the 5-year survival is less than 5% [1]. Surgical resection offers the best chances of survival at the time of the diagnosis, but is curative in only 13% of the cases and is possible for only 15% of the patients. Moreover, even resectable PDACs display a high rate of recurrence [1]. Systemic chemotherapy still relies on the pyrimidine analog gemcitabine because it has been the only drug producing symptoms improvement, raising the overall 1-year survival from 2% to around 18% of the patients. Extensive efforts have been made to identify adjuvant or neoadjuvant therapies capable of improving the poor prognosis of PDAC, based on the molecular targets involved in cancer progression. Unfortunately, phase III studies have shown limited or even no improvement in patient survival in combination with gemcitabine [1]. In fact, PDAC presents very complex genetic alterations profiles explaining the failure of single gene/pathway targeted adjuvant therapies. Indeed, the Pancreatic Cancer Genome project has analyzed 23,219 transcripts and identified an average of 63 somatic mutations per PDAC affecting 12 core signaling pathways and the overexpression of 500 different genes in 24 tumors [2].

Thus, it is now critical to test therapies targeting several pathways or therapies inducing specific tumor cell death [3]. In that aspect, cytoreductive therapy inducing direct cell death rather than corrective therapy aimed at repairing genetic defects involved in malignancy should be preferred for PDAC, because of the general resistance of tumor cells against therapeutic agents. In that regard, gene therapy remains an attractive option to transfer suicide genes [4-6]. Specific efficient expression of suicide genes in tumor cells is currently achieved by designing vectors containing tissue-specific promoters. Moreover, suicide genes are chosen to be able to induce bystander killing of cancer cells in the vicinity of gene-modified (transduced) cancer cells [5]. Disappointing results however, have been obtained in phase I/II trials with adenoviruses or retroviruses, but studies are still focusing on improving this treatment conditions [3]. Over the numerous means to perform *in vivo* gene delivery, increasing interest has been shown in lentiviral vectors because they can infect non-dividing cells and display high gene delivery efficiency. Previous studies have shown the feasibility of gene therapy in PDAC [7-9] with diverse efficiencies of gene transfer. Lentivectors are commonly packaged into viral envelopes exhibiting glycoproteins with a broad tropism, such as Vesicular Stomatitis Virus-Glycoprotein (VSV-G). It is however important to restrain the infectious capacity of therapeutic vectors to the tumor cells only, limiting side effects on the neighboring normal cells. Cell-specific targeting has been developed using a modified envelope displaying the IgG binding-domain of protein A, which can bind the Fc domain of immunoglobulins. In consequence, virions can be associated with cell surface-directed antibodies to target the specific transduction of cancer cells [10-12]. This system has given good results with *in vivo* models to transduce prostate cancer bone metastasis [13], the therapeutic gene thymidine kinase in prostate cancer metastasis [14] and breast cancer cells [15].

To date, the possibility of specific gene transfer in PDAC tumor cells has not been evaluated, and this study was aimed to test the modified Sindbis virus glycoprotein to target PDAC cells *in vivo*. PDAC cell surface antigens have been selected according to published data. Recently, a list of biomarker candidates for PDAC has been obtained from a comprehensive literature survey [16]. Accordingly and as previously reported [17,18], two markers appeared appropriate for the present study because of their localization at the cell surface and their specific overexpression in PDAC, namely the variant 2 of Claudin-18 (CLDN18) and the Mucin-4 protein (MUC4). The Prostate Stem Cell Antigen (PSCA) was also included because it was used by Pariente et al. to target prostate cancer cells with the same system [13] and has been proposed for immune therapy [19].

In this study, specific reporter gene transfer has been examined *in vitro* with antibodies directed against the cell surface markers described above. More importantly, *in vivo* targeting of pancreatic cancer cells has been tested in subcutaneous and orthotopic xenografts models and quantitatively compared to a broad tropism virus. Our models included the use of a grafted PDAC cell line modified to stably express the tdTomato reporter gene, which expression was directly monitored in live animals by fluorescence detection. Finally, the possibility of transferring the suicide gene thymidine kinase has been assessed in orthotopic xenografts, which growth was monitored by the detection of tdTomato.

## Results

### Targeted transduction of pancreatic cell lines *in vitro*

**Figure 1:**
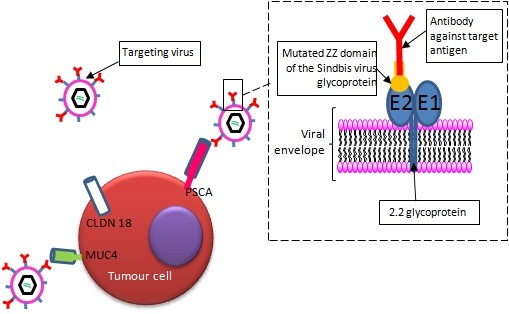
**Oncospecific transduction in human pancreatic tumor cells.** PDAC cell surface specific antigens were chosen according to previous reports. They were used to target lentiviral transductions of the Green Fluorescent Protein (GFP) reporter gene, using a Sindbis virus modified glycoprotein. The E2 recognition ZZ domain of glycoprotein has been modified to contain the Fc-binding domain of the protein A. The resulting mutant glycoprotein was called 2.2. Thus, virions can be associated with cell surface-specific antibodies to target the transduction of tumor cells (insert).

To package the lentiviruses, we used a modified Sindbis virus glycoprotein. The E2 recognition ZZ domain of the Sindbis virus glycoprotein has been modified to contain the Fc-binding domain of the protein A [12]. The resulting mutant glycoprotein was called 2.2 [13,14]. Thus, virions can be associated with cell surface-specific antibodies to target the transduction of tumor cells (Figure 1). They will be referred as oncotropic. Transductional targeting was performed using antibodies that recognize the PSCA (Prostate stem cell antigen), the MUC4 and the Claudin 18 variant 2 antigens (CLDN18), all chosen according to published data showing specific expression or overexpression of these proteins in pancreatic adenocarcinomas (see Background section, Figure 1).

**Figure 2:**
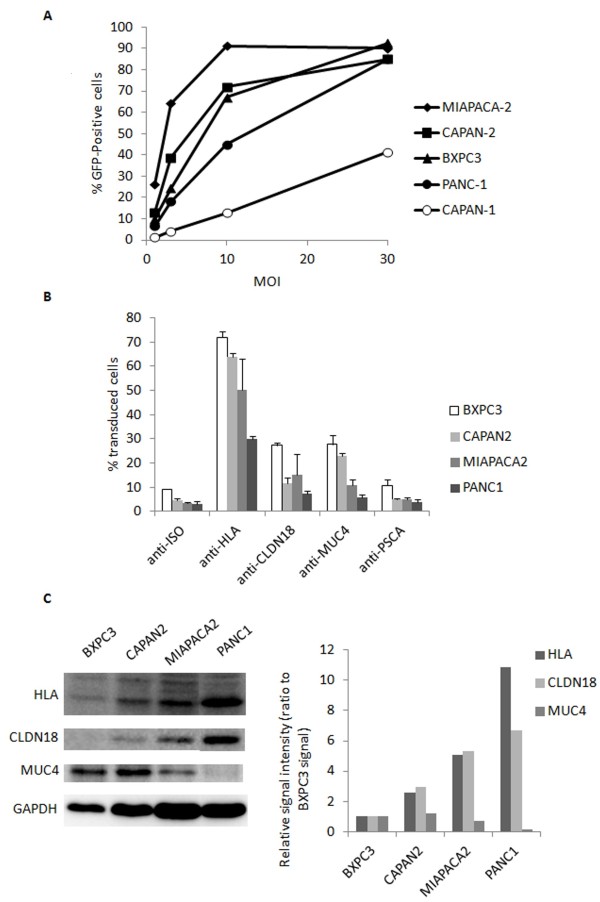
**Targeted-transduction of human pancreatic cell lines by the specific pseudotyped GFP-expressing lentivirus. A)** PDAC cell lines were transduced with lentiviruses packaged into VSV-G-containing envelopes at increasing multiplicity of infection. MOI = 10 corresponds to a p24 = 100 ng. **B)** PDAC cell lines were transduced with 100 ng of p24 oncospecific lentiviruses packaged into the 2.2 Sindbis virus glycoprotein-containing envelopes bound to the antibodies mentioned on the x axis (0.5 μg). The y axis corresponds to the% of transduced cells expressing the reporter gene GFP analysed by flow cytometry. Each condition has been tested at least in 3 independent experiments. All conditions with oncospecific lentiviruses are statistically significant (Student’s t test, p < 0.01) compared to negative control condition (ISO) except for PSCA-targeting viruses. **C)** Western-blots of PDAC cell lines protein extracts with the antibodies used for the targeted transduction. Anti-GAPDH was used as a loading control. After quantification of the western blots by densitometry, the levels of protein expression were calculated as a ratio of GAPDH levels and are shown in a bar graph as a ratio to levels observed in the BXPC3 cell line.

To test the capacity of pancreatic adenocarcinoma cell lines to be transduced, we performed lentiviral transductions with a VSV-G pseudotyped lentiviral vector carrying a transgene encoding the reporter gene green fluorescent protein (GFP). Multiplicities of infection ranging from 1 to 30 (MOI1 to MOI30, corresponding to 10 ng of p24 to 300 ng of p24 for 5 × 10^4^ cells, p24 being a viral capside protein titrated as described in the Methods section) were used and cells were analyzed by flow cytometry for GFP expression 4 days after the transductions. All the cell lines were transduced by the lentiviruses with various efficiencies (Figure 2A). The CAPAN1 cell line was, in our conditions, more resistant to transduction than the other cell lines tested, since the percentage of GFP-positive cells did not reach more than 40%, at the highest MOI used. Thus, we discarded this cell line for the subsequent experiments. PANC1 cells seemed moderately resistant to viral transduction.

The pancreatic cancer cell lines were then transduced with the oncotropic vectors (with 100 ng of p24 for 5 × 10^4^ cells). They were also transduced with an oncotropic lentivirus associated to an anti-HLA antibody as a positive control or associated with isotype control antibodies (rabbit IgGs) as a negative control. Cell lines displayed efficient transduction with the positive control since at least 30% of the cells were transduced by the viruses associated with the anti-HLA antibody (30% for the PANC1 to 71% for the BXPC3, Figure 2B). Furthermore, the use of anti-PSCA antibody did not achieve transduction efficiencies above that of the negative control values in all the tested lines. Conversely, values with anti-MUC4 and anti-CLDN18 reached percentages of transduction at least 2 times above that of the negative control, the best results being for the anti-CLDN18 in the MIAPACA2 cells and the anti-MUC4 in the CAPAN2 cells (5 times). We asked whether the differences in transduction efficiencies were dependent on the levels of the target surface antigens. Western-blots analyses showed that the PANC1 cell line expressed the highest level of HLA proteins (Figure 2C), but was the least efficient in transduction (only 30%, Figure 2B) as already observed with VSV-G pseudotyped lentivectors (Figure 2A). On the other hand, the MIAPACA2 cells had a very good potential for transduction with the broad tropism virus (Figure 2A), but showed a weaker efficiency with the MUC4 oncotropic virus as compared with the BXPC3 and CAPAN2 cells. Western-blot analysis showed that the MIAPACA2 did express lower levels of MUC4. Therefore, the efficiency of transduction seems to depend on both the intrinsic ability of the cells to be transduced and the level of expression of the cell surface antigen targeting the oncotropic lentivirus.

Thus, the anti-CLDN18 and anti-MUC4 antibodies displayed sufficient transductions *in vitro* to be further evaluated in *in vivo* xenograft models with the CAPAN2 and the MIAPACA2 cells.

### Targeted transduction of pancreatic cell lines *in vivo*

**Figure 3:**
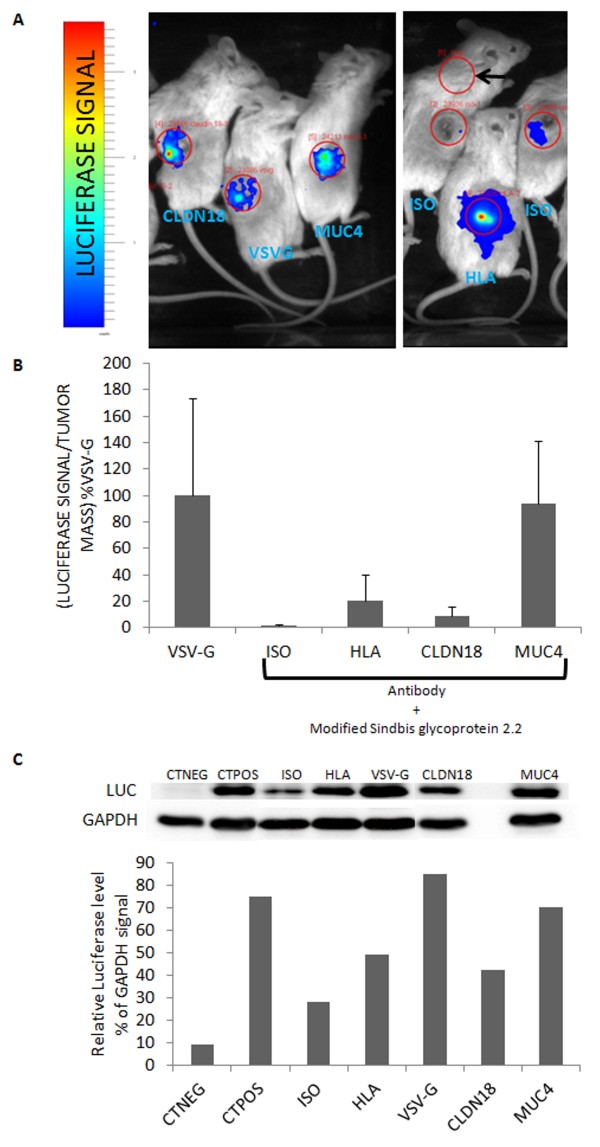
**Anti-MUC4 antibodies bound to modified lentiviruses drive efficient gene transfer *in vivo *in CAPAN2 cells. (A)** CAPAN2 cells were grafted subcutaneously in the right flanks of immune-deficient recipient mice. When tumors reached about 100 mm^3^, lentiviruses carrying the luciferase reporter gene combined with anti-MUC4, anti-CLDN18 (PDAC targeting antibodies), anti-HLA (positive control for the targeted gene transfer), rabbit IgGs (ISO, negative control for the targeted gene transfer) or packaged into the broad tropism VSV-G-containing envelope (positive control) were directly injected in the tumors (n = 3 in each group). Two weeks after virus injections luciferase signal was measured in anesthetized live animals with a photon imager apparatus. **(B)** Quantification of luminescence in photons/steradiant/s (ph/sr/s) was divided by tumor mass in mg, since tumor mass could be very different in individuals after resection, and is expressed as a percentage of VSV-G positive control. Red circles depict the zones used for quantification (same size for all the mice). The black arrow points the area measured for background signal determination. Student’s t tests against the VSV-G condition were statistically different for ISO (p = 0.04) and CLDN18 (p = 0.05). **(C)** Western-blot analysis of luciferase expression in the previous tumor extracts. The negative control (CTNEG) corresponds to a tumor extract of untransduced CAPAN2 cells. The positive control (CTPOS) was obtained from a tumor derived from CAPAN2 cells transduced with a lentivirus bearing the bicistronic transgene LUCIFERASE-IRES-ZsGREEN. Extracts obtained from tumors transduced by oncospecific lentiviruses are identified according to the target cell surface antigen. ISO: rabbit IgGs.

To assess the efficient transduction of the cells *in vivo*, the lentiviruses were designed to express the firefly luciferase, for which expression was detected in live animals by a bioimaging system. In order to test the oncotropic gene transfer in pancreatic tumor cells *in vivo*, CAPAN2 cells were xenografted subcutaneously in immune-compromised mice. Anti-MUC4 and anti-CLDN18 antibodies were conjugated to lentiviruses packaged into an envelope containing the modified 2.2 Sindbis virus glycoprotein and injected directly in the tumors. Control mice were injected with anti-HLA (positive control) or rabbit IgGs (negative control) conjugated viruses. Moreover, a group of tumor bearing mice was injected with broad tropism viruses packaged into the VSV-G-containing envelopes (positive control), since it was previously shown to cause efficient gene transfer in pancreatic adenocarcinoma [7-9]. Luciferase signals were evaluated according to the tumor mass, since all tumors were not equivalent within each group, and in all groups. As expected, two weeks after the virus injections, tumors injected with rabbit IgGs-conjugated viruses showed gene transfer less than 2% of VSV glycoprotein-containing viruses and the association with anti-HLA represented about 20% of the signal obtained for VSV-G-containing envelope (Figure 3A,B). Very interestingly, viruses conjugated with anti-MUC4 were as efficient as the broad tropism viruses (93% of the signal). Anti-CLDN18 was less efficient to target the CAPAN2 cells since only about 9% of the VSV-G signal was obtained (Figure 3). Gene transfer efficiency was further examined in resected tumors by detection of the luciferase protein by western-blot (Figure 3C). We used a tumor extract derived from untransduced CAPAN2 cells as a negative control (CTNEG) and a positive control (CTPOS) obtained from a tumor derived from CAPAN2 cells transduced with a lentivirus bearing the bicistronic transgene Luciferase-IRES-ZsGreen. Before the grafts, cells were sorted and at least 80% expressed the reporter protein ZsGreen. Quantitatively, the levels of luciferase in the positive control were less than in the tumors transduced with the broad tropism virus (Figure 3C, CTPOS versus VSV-G). In the positive control however, the luciferase open reading frame (ORF) was behind the IRES, whereas in the “VSV-G” condition, the luciferase ORF is first, because different lentivectors were used. Thus, it was expected to obtain a lower expression level in the CTPOS. Comparatively, the efficiencies of transduction seemed very good with MUC4 oncotropic viruses, and a little less with the HLA and CLDN18 targeting lentiviruses. Overall, the western-blot confirmed the results obtained by direct luciferase activity detection in the tumors.

**Figure 4:**
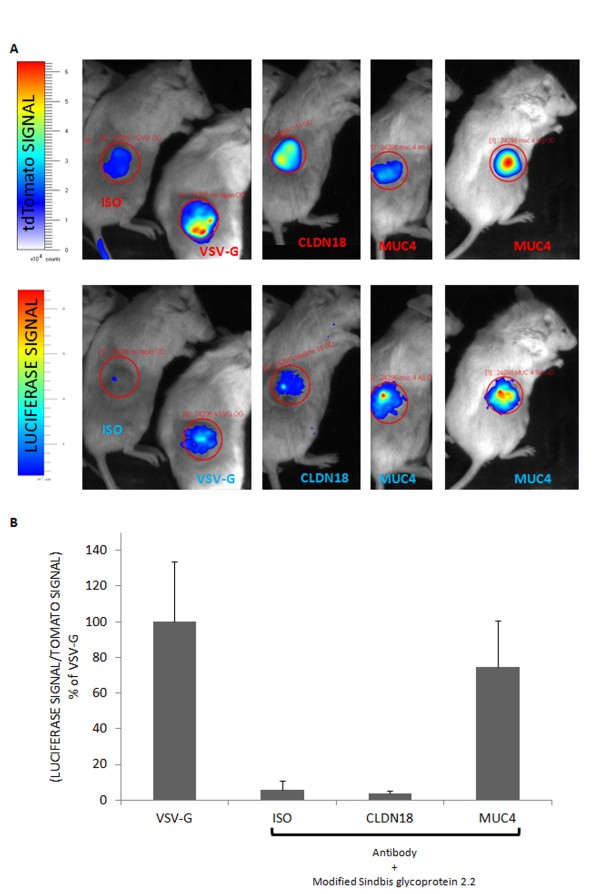
**Luciferase expression driven by MUC4-oncotropic virus co-localizes with fluorescence in tdTomato-MIAPACA2 tumors. (A)** MIAPACA2 cells were modified to express permanently the tdTomato reporter gene, detectable with the photon imager in subcutaneously grafted tumors (top panel). Lentiviruses carrying the luciferase reporter gene combined with anti-MUC4, anti-CLDN18 (PDAC targeting antibodies), rabbit IgGs (ISO, negative control for the targeted gene transfer) or packaged into the broad tropism VSV-G-containing envelope (positive control) were directly injected in the tumors (n = 3 in each group). Three weeks after virus injections luciferase signal was measured in anesthetized live animals with a photon imager apparatus (bottom panel) and co-localizes only with fluorescent tumor cells. **(B)** Quantification of luminescence in ph/sr/s was divided by tumor fluorescence (ph/sr/s) and is expressed as a percentage of VSV-G positive control. Student’s t tests against the VSV-G condition were statistically different only for ISO CLDN18 (p = 0.05 for both). Red circles depict the zones used for quantification (same size for all the mice).

To further explore targeted gene transfer in PDAC, another cell line was tested. First, the MIAPACA2 cells were modified to stably express the fluorescent protein tdTomato for the direct follow up of tumor progression in live animals using the bioimaging system, and the quantification of tumor growth. In subcutaneous tumors (Figure 4), the anti-CLDN18 antibody failed to raise the signal above that of the isotype control. However, anti-MUC4 conjugated viruses were again almost as efficient as viruses packaged into the VSV-G-containing envelope (about 75% of the “VSV-G” signal). This result was confirmed by using two different anti-MUC4 antibodies.

Thus, in subcutaneous tumors, intra-tumoral injection of lentiviruses associated with the tumor cell surface antigen MUC4 seemed efficient enough to test the transfer of a suicide gene.

### Targeted transduction of the herpes simplex virus thymidine kinase gene is toxic in pancreatic cell lines *in vitro* and *in vivo*, in the presence of ganciclovir

**Figure 5:**
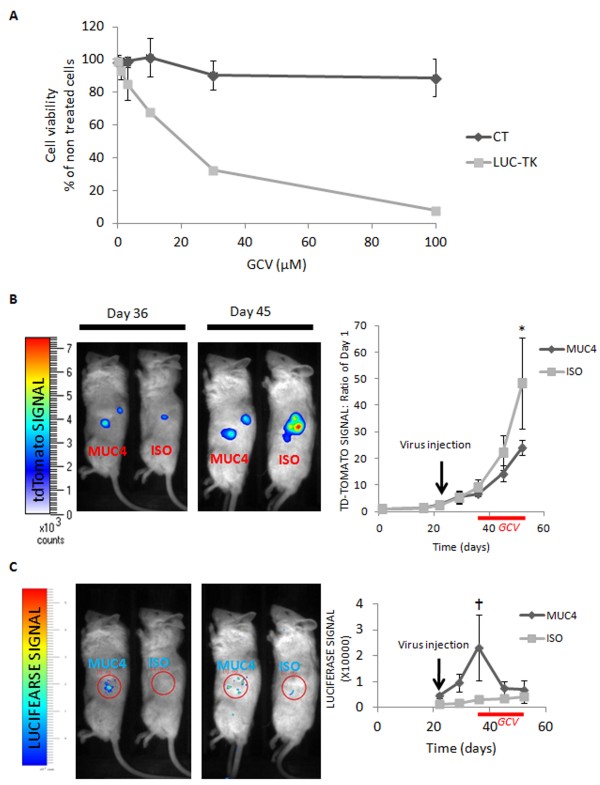
**Targeted transduction of the Herpes Simplex virus thymidine kinase gene is toxic *in vitro *and *in vivo*, in the presence of ganciclovir. A)** MIAPACA2 cells stably expressing the tdTomato protein were transduced with viruses packaged into broad tropism envelopes, expressing either the GFP protein (CT, dark gray line) or a bicistronic gene encoding the firefly luciferase and the herpes simplex virus thymidine kinase (LUC-TK, light gray line). Cells were treated with ganciclovir. Cell death was examined 10 days after treatment. Cell viability was measured by MTS tests. ***: p<0.001 in Student’s t test compared to control condition. **B)** TdTomato-MIAPACA2 cells were grafted in the pancreas of recipient mice. After 21 days anti-MUC4-conjugated viruses (MUC4) or control IgGs-conjugated viruses (ISO) were injected in the intra-pancreatic tumors (n=6 animals in each group). After 36 days, ganciclovir was injected daily for two weeks (solid red lines). Left panels: fluorescence corresponding to grafted tumor cells the first day of GCV injection (Day 36) and one week after the beginning of GCV injections (Day 45). Right chart: Quantification of fluorescence signals expressed as a fold increase of fluorescence detected on day 1. **C)** Left panels: luminescence signals observed in mice the first day of GCV injection (Day 36) and one week after the beginning of GCV injections (Day 45). Right chart: Raw luminescence signals are reported as ph/sr/s from the day of virus injection (day 21) to the day of experiment termination (day 45). On day 21, when viruses were injected, we had lost 1 mouse in the MUC4 group and 2 mice in the ISO group. On day 45, we had only 3 mice in each group. p=0.06 with a 2-sided Mann Whitney test against control signal, n=3. * p=0.018 with a 2-sided Mann Whitney test against control signal, n=4-5.

Since we produced MIAPACA2 cells stably expressing the tdTomato fluorescent protein, we first checked that these cells were sensitive to ganciclovir when expressing the Herpes Simplex virus thymidine kinase gene. Cells were transduced with viruses packaged into broad tropism envelopes (from VSV-G), expressing either the GFP protein (CT) or a bicistronic gene encoding the firefly luciferase and the Herpes Simplex virus thymidine kinase (LUC-IRES-TK). Cells were treated with increasing concentrations of ganciclovir and cell viability was examined 10 days after the treatment (Figure 5A). Expectedly, cells expressing the thymidine kinase died in the presence of ganciclovir in a dose-dependent manner.

**Figure 6:**
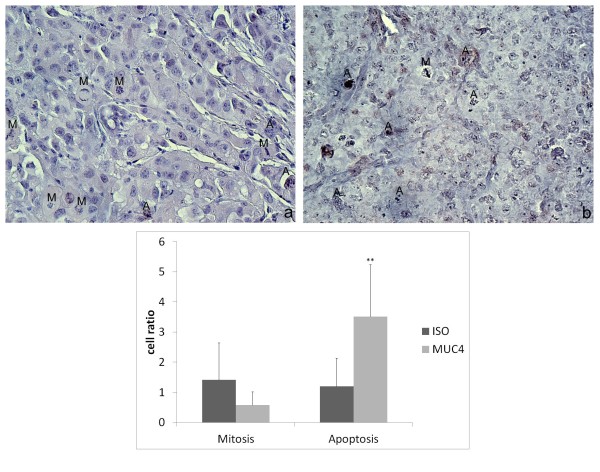
**Mitotic and apoptotic figures on sections from ISO and MUC4-oncotropic lentivector-injected tumors.** Sections from tumors injected with ISO-oncotropic control lentivector **(A)** or with MUC4-oncotropic lentivector **(B)** were stained with hematoxilin. Mitotic figures (**M**) and apoptotic figures (**A**) were counted in 2791 (ISO) or 1558 (MUC4) cells (n = 3). **: p = 0.002 with Student’s t test. Original magnification X40.

TdTomato-MIAPACA2 cells were grafted in the pancreas of recipient mice. Tumor growth was monitored by fluorescence detection for 21 days (Figure 5B). MUC4-oncotropic viruses or control IgGs-conjugated viruses were then injected in the intra-pancreatic tumors. After about 2 weeks, luminescence signals were very high in the “MUC4” injected mice (Figure 5C), suggesting efficient gene transfer in the tumor cells (p = 0.018 with a 2-sided Mann Whitney test against control signal, n = 4-5). Mice were then treated with ganciclovir (GCV) daily, and the experiment was terminated by sacrificing the mice 2 weeks after the first injection of GCV. GCV injection induced a drop of luminescence signal in the “MUC4” mice as soon as 7 days after the beginning of the treatment (Figure 5C). The signal then remained low, and up to the level of that of the control mice. Concomitantly, when tumor growth went on to be about 50 times bigger than at the beginning in the control mice, tumor growth was slowed down in the “MUC4” mice and reached only about 24-fold of the initial fluorescence value (Figure 5B p = 0.06 with a 2-sided Mann Whitney test against control signal, n = 3, our initial group of 6 mice in each group having dropped by half because of surgery complications or tumor development). Mitosis and apoptotic figures were quantified on tumor sections. While the number of cells in M phase was not different between tumors, tumors that had received the oncotropic lentiviruses displayed higher numbers of apoptotic figures (Figure 6).

**Figure 7:**
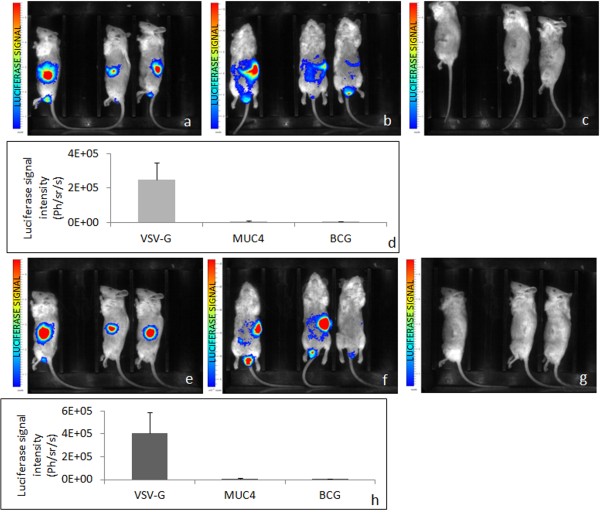
**Oncotargeted transduction of lentiviruses is efficient only in pancreases bearing tumors.** Ten μg of p24 lentiviruses carrying the luciferase reporter gene packaged into the VSV-G-containing envelope **(A** lateral view, **B** ventral view) or anti-MUC4-conjugated lentiviruses **(C** lateral view) were directly injected in the pancreas of tumor free animals (n = 3 animals in each group). Luciferase signal was visualized after one week. Luciferase signal intensity was quantified and is reported as mean of Ph/sr/s ± SEM **(D)**. Increased luciferase signal is shown after 16 days in the same animals (**E,F:** VSV-G and **G:** MUC4), with signal quantification **(H)**.

Lentiviruses were injected intra-tumorally and there was still a possibility that they transduced the tumors just because they were provided locally at a high concentration. To test this hypothesis, we performed intra-pancreatic injections of the broad tropism virus and of the MUC4 oncotropic virus in tumor-free mice. After one week, the broad tropism virus transduction was highly detectable in the pancreas (Figure 7A) but also the virus had leaked to transduce other tissues such as the liver, the intestine and surprisingly the region of the testis (Figure 7B). Conversely, the MUC4 oncotropic virus did not achieve any detectable transduction in the pancreas or in any other site (Figure 7C). After two weeks, the broad tropism viral transductions were still detectable with higher signals in the pancreas (Figure 7E) and had been partly cleared from the liver, but remained and even increased in the gut and the testis. No new signal appeared in the MUC4 oncotropic virus-injected mice.

Thus intra-tumoral injections of the MUC4 oncotropic virus appeared to be a very specific way to perform gene delivery in pancreatic tumors by contrast to a broad tropism virus, which transduced normal pancreatic cells and reached tissues distant from the tumor and the injection site.

## Discussion

In this study, we tested for the first time the possibility to specifically deliver a therapeutic gene into pancreatic tumor cells using cell surface markers.

Gene transfer with lentiviruses is a very potent approach of gene therapy since viruses can transduce cells regardless of their stage in the cell cycle [20]. Previous studies have reported the possibility to use lentivectors for gene delivery to pancreatic cancer cells but were performed with broad tropism envelopes [7-9]. This approach might represent a risk of morbidity because of possible integrations of the viruses in normal cells (off target transductions). However, viral gene therapy still holds the promise of oncotargeting gene delivery sparing the normal cells, thanks to the modified Sindbis virus glycoprotein developed by Pariente et al. [13] and used here. This approach offers the possibility to specifically pseudotype the viral particles. We found that modified Sindbis virus glycoprotein-packaged lentiviruses could efficiently transduce the pancreatic cell lines *in vitro*. Three pancreatic cell surface antigens were tested for specific lentiviral gene transfer. The use of anti-PSCA yielded very poor gene transfer, regardless of the cell line. This result was disappointing since this protein was found highly expressed in pancreatic adenocarcinoma [21] and anti-PSCA drove efficient gene transfer in prostate tumors [13]. Interestingly however, CLDN18 and MUC4 achieved the best results in terms of efficiency and specificity *in vitro* and could be suitable for specific gene transfer in pancreatic tumor cells. To test this hypothesis, human tumor cells were grafted under the skin of immune-deficient mice. Therapeutic agent administration by intra-tumoral injections is possible in pancreatic tumors since it has been performed in clinical trials with endoscopic ultrasound injections [22]. Moreover, intra-tumoral injection of therapeutic oncotropic lentiviruses might be safer than intra-venous delivery to limit any systemic toxicity. Anti-MUC4-pseudotyped viruses carrying the firefly luciferase reporter gene, directly injected in the tumors yielded, luminescence signals in the tumors comparable to signals obtained with viruses packaged into the non-specific envelope containing the VSV glycoprotein, in two different cell lines. *In vivo*, MUC4 was a more potent antigen than CLDN18 and even than HLA antigens in the CAPAN2 cells. Remarkably, luminescence appeared confined to the tumors since no signal was detected elsewhere in the mice, even when the detection mode was used with very low stringency. This observation was made previously when the gene transfer targeting system used here was tested by others [13-15]. Noteworthy, pancreatic injections of viruses in tumor-free mice led to very interesting observations. First, the broad tropism virus transduced very efficiently the pancreas and leaked in other intra-abdominal sites, and even in the testis. By contrast the MUC4 oncotropic virus showed no detectable transduction at any site when injected in the pancreas, in the absence of tumors, even at the injection site. Taken together, this set of data suggests that the oncotropic lentiviral transductions appeared safer and more specific than VSV-G-driven transductions.

The results with CLDN18 oncotropic viruses were somewhat disappointing in the *in vivo* transductions, considering that the fact that similar results were obtained for CLDN18 and MUC4 oncotropic viruses *in vitro* (Figure 2B). We actually noticed strong signals one week after virus injections with anti-CLD18 antibodies, but signals had partially disappeared in CAPAN2 and almost totally disappeared in MIAPACA2 cells at the time of sacrifice, after two weeks (not shown). One possible explanation could be that fixation of anti-CLDN18 might interfere with the biological function of claudin 18 in cancer cells, probably leading to cell death.

Herpes thymidine kinase (TK) in combination with the pro-drug ganciclovir remains one of the most potent systems for anticancer gene therapy approach and has given promising results in a very recent phase I clinical trial with an adenoviral system [6]. We evaluated the transfer of the TK gene by MUC4 oncotropic lentiviruses injected in orthotopically grafted human pancreatic tumor cells. Our experimental strategy was designed to do both the follow up of tumor growth (by fluorescence) and of the virus-infected tumor cells (by bioluminescence) in live animals. Importantly and as observed before, luminescence remained confined to the tumors when viruses were injected directly in the pancreas of the recipient mice. Moreover, GCV treatment resulted in luciferase signal loss and in slowing down of the tumor growth. It would be worth now to use this strategy to examine other PDAC-specific cell surface targets, and we feel that this study presents the proof of concept of oncotargeted molecular therapy of PDAC. There are many ways to improve the system. First, several targets (cell surface markers) could be used in concert as well as several rounds of virus injections could be performed to gain in efficiency. Second, once the markers have been validated, it is now possible to use vectors pseudotyped with engineered Sindbis virus glycoprotein bearing a covalent link with the antibodies. Indeed, fusion proteins could be produced [23] rendering the transduction very potent even in an immune-competent background. Another attractive option would be the use of the biotine/avidine combination developed more recently [24].

## Conclusion

Our study outlines for the first time three major concepts: (i) lentiviral transduction of human pancreatic tumor cells was possible when cells were grafted directly in the pancreas, (ii) this transduction was achieved with a system targeting the tumor cells with cell surface antigens, sparing the normal cells and (iii) detectable loss of reporter gene-expressing cells obtained by viral transduction was observed with the TK/GCV anticancer system. Moreover, the approach presented here appeared to be a safer, much more specific and an as efficient way to perform gene delivery in pancreatic tumors, in comparison with a broad tropism lentivirus. Importantly, we have developed an orthotopic graft model of human PDAC allowing the quantification of tumor growth and the co-localization of oncospecific targeting with direct, costless and non invasive procedures. Future improvement of this gene therapy approach includes the identification of other potent cell surface markers, the use of combinatory cell surface markers for specific gene transfer and the development of oncotropic envelopes stable in immune competent background.

## Methods

### Animals, pancreatic cell lines and antibodies

The NOD/Shi-SCID IL2R^γnull^ mice were produced and housed in the University Bordeaux Segalen animal facility, according to the rules and regulations governed and enforced by the Institutional Animal Care and Use Committee. The animal facility institutional agreement number is A33063916. Animals were included in protocols between 6 and 8 weeks old. Mice were monitored weekly for body weights and were also examined for aspect and behavior during the time-course of the experiments. No changes were noticed except otherwise indicated. The PDAC PANC1 and MIAPACA2 cell lines were purchased from the ATCC (American Type Culture Collection, Molsheim, France). CAPAN2 and BXPC3 were kindly provided by Joel Tardive-Lacombe (INSERM U624, Marseille, France). The CAPAN2 and BXPC3 cells were maintained in RPMI (Invitrogen) with 10% Fetal Calf Serum (FCS, Invitrogen) and Penicillin/Streptomycin 1/100 (Invitrogen), the PANC1 and MIAPACA2 were cultured in DMEM with 10% FCS and Penicillin/Streptomycin 1/100.

The tdTomato-MIAPACA2 cell line was produced by transduction of the MIAPACA2 cells with a lentivirus carrying the tdTomato reporter gene (PGK-tdTomato, see below). Transduced cells were sorted by a BD FACS ARIA cell sorter (BD Biosciences, France).

The antibodies used in this study were purchased as follows: anti-HLA, anti-MUC4, rabbit IgGs (SIGMA ALDRICH, Lyon, France), anti-CLDN18 (GenWay, San Diego, CA-USA), anti-PSCA (Abcam, Paris, France), anti-PSCA (SIGMA ALDRICH, Lyon, France).

### Vector construction, production and transduction of cells

pPGK-tdTomato lentiviral plasmid was constructed by transferring the tdTomato gene from p-tdTomato (Clontech, Saint Germain en Laye, France) into pRRL-Sin-cPPT.PGK.WPRE lentiviral plasmid (gift from Dr. Trono, Lausanne, Switzerland). LUCIFERASE-IRES-TK lentivirus plasmid was obtained by replacing GFP from pLOXgfp-IresTK (Addgene Plasmid # 12243, Cambridge, MA-USA) with the firefly-Luciferase gene (Clontech). Cloning details can be provided upon request. A LUCIFERASE-IRES-ZsGreen lentivirus plasmid was obtained as follows: the fireflyLuciferase gene was cloned into the pIRES2-ZsGreen1 vector (Clontech, Saint Germain en Laye, France). All lentiviral vectors were produced by calcium phosphate mediated triple transient transfection of 293 T cells with one of the vector transfer constructs, the packaging construct pCMVΔ8.91 (gift from Dr. Verma, La Jolla, CA-USA) and either VSV-G construct psPAX2 (gift from Dr. Trono) or 2.2 plasmid (a gift from Drs. Chen and Morizono, coding for a modified Sindbis virus glycoprotein envelope, Los Angeles, CA-USA). The viruses produced were concentrated by ultracentrifugation (through a 10% sucrose cushion). The capside protein p24 titrations were determined as already described [25].

### Analysis of lentiviral transduction by flow cytometry

FACS analyses were performed on a FACScalibur flow cytometer (BD biosciences, Le Pont de Claix, France) on trypsinized cells 3–5 days after transduction. Transductions were carried out on 5.10^4^ cells in 48-well plates. Virus mixes containing 100 ng of p24 were prepared in 250 μl of serum-free medium with antibodies at 0.5 μg/ml. Percentages of GFP-positive cells were determined using CellQuest software (Becton Dickinson, Le Pont de Claix, France) in comparison with non transduced cells, after counting of cells in the FL-1 channel.

### Western-blots

Protein extracts were prepared in RIPA buffer and processed for western blotting. Membranes were incubated with the targeting antibodies or a rabbit anti-luciferase antibody (AbCam, Paris, France). Rabbit anti-GAPDH antibody (Cell Signaling Technologies, Saint-Quentin-en-Yvelines, France) was used to assess equal loading of the samples. Primary antibodies were detected with specific anti-rabbit- or anti-mouse-IgG-HRP (Cell Signaling Technologies). Proteins were visualized using the ECL detection system (Amersham Pharmacia Biotech, Orsay, France). Quantification by densitometry was performed with the ImageJ software.

### Histology

Tumors were fixed in 10% NBF, embedded in paraffin and processed by routine histology procedures. The proportion of mitosis/total cells or apoptosis/total cells was evaluated after Hematoxilin staining by direct counting on pictures taken at X40 magnification.

### *In vitro* cell proliferation assay

To test the effect of Herpes Simplex virus thymidine kinase on the tdTomato-MIAPACA2 cell viability in the presence of ganciclovir (GCV, SIGMA ALDRICH, Lyon, France), cells were first transduced with a lentivirus bearing the LUCIFERASE-IRES-TK transgene (see above). Cells were plated at 3.10^3^ cells per well in 96-well plates. The day after, increasing doses of GCV (0-100 μM) were applied and cells were kept in culture for 10 days. Each point was done in quadruplet. The experiments were carried out 2 times. Cells were washed with PBS and treated with MTS (3-(4,5-dimethylthiazol-2-yl)-5-(3-carboxymethoxyphenyl)-2-(4-sulfophenyl) - 2H-tetrazolium) solution (Promega, Charbonnières, France) for 1.5 h to determine cell viability by reading optical densities (OD) at 490 nm. Results are expressed as cell viability: $\left({\text{OD}}_{\text{treated}}\right)/{{\text{OD}}_{\text{non}}}_{\text{treated)}}\times 100.$

### Bioluminescent imaging of pancreatic adenocarcinoma xenografts

For subcutaneous xenografts, groups of 3–5 mice were anesthetized with isoflurane. 8.10^5^ to 10^6^ cells in 100 μl medium were injected in the right flanks. When tumors reached about 100–150 mm^3^, diverse combinations of viruses (described in the result section) were injected in the tumors or directly in tumor-free pancreases in single injections. Briefly, 7 μg p24 of lentivirus were mixed, when necessary, with 5 μg of antibody and incubated 5 min at room temperature in 60 μl serum free-medium. Tumors were monitored for luciferase expression on a weekly basis as follows. 150 mg/kg of D-Luciferin (Interchim, Montluçon, France) was injected intraperitonally. After 10 min, mice were anesthetized by isoflurane and placed into a photon bioimager (BIOSPACE LAB, Paris, France) for about 20 min to acquire luminescence images. Signals were quantified with the M3Vision software (BIOSPACE LAB).

For orthotopic xenografts, groups of 4–6 mice were anesthetized with isoflurane. Pancreases were exposed and 8.10^5^ of tdTomato-MIAPACA2 cells in 40 μl medium were injected directly in the pancreas. Tumor growth was monitored weekly with the bioimager using the fluorescence detection setting (Acquisition mode: FLI integration at 22 ms per frame, Excitation = 520 nm, Background = 480 nm, Emission = 570 nm, Filter cut off = 570 nm Illumination: 100%). When signals were easily detectable (after 21 days), mice were anesthetized with isoflurane and injected with the lentiviruses. Ten μg p24 of lentiviruses were incubated 5 min at room temperature in 60 μl serum free-medium with 5 μg of antibody and injected intra-tumorally as described above. Luciferase expression and fluorescence signal were monitored weekly. Two weeks after virus injections, mice were treated daily with GCV (1 mg/mouse).

### Statistics

Transduction efficiencies *in vitro* are expressed as mean% of transduced cells ± SD. MTS assay results and *in vitro* transduction results are expressed as mean ± SD. Luminescence quantifications performed *in vivo* are expressed as mean ± SEM. Statistical tests were performed with Student’s t tests or with a 2-sided Mann Whitney test for intra-pancreatic luciferase detections and tumor growth.

## Competing interests

The authors declare that they have no competing interests.

## Authors’ contributions

ML, MT and SD carried out *in vitro* assays. Western blotting was performed by IM. *In vivo* experiments were designed by FMG, PD, EP and SD and were carried out by ML, BR, IM and SD. VGD, MT and FMG designed and produced the vectors. ML, BR, FMG and SD analyzed the results and produced the figures. EP, HV and AB participated in the discussion and interpretation of the study and manuscript preparation. SD and FMG wrote the manuscript. All authors read and approved the final manuscript.
